# Applying metacognitive therapy for generalized anxiety disorder to adolescents: results from an open trial with 3- and 6-months follow-up

**DOI:** 10.3389/fpsyt.2026.1765814

**Published:** 2026-05-05

**Authors:** Hanne Undheim Hoff, Odin Hjemdal, Henrik Nordahl

**Affiliations:** 1 Department of Psychology, Norwegian University of Science and Technology, Trondheim, Norway; 2 St. Olavs Hospital, Division of Child and Adolescent Psychiatry, Avdeling for barne- og ungdomspsykiatri (BUP), Trondheim, Norway

**Keywords:** adolescents, generalized anxiety, mechanisms, metacognitive therapy, worry

## Abstract

**Introduction:**

In adults, Metacognitive therapy (MCT) for Generalized anxiety disorder (GAD) has demonstrated to be an effective treatment, and even superior to comparisons in randomized controlled trials. One explanation for these results can be that MCT directly modifies dysfunctional metacognitive beliefs (including beliefs about worry as uncontrollable) which in the metacognitive model are assumed to be the underlying mechanisms in excessive worrying and emotional distress. However, MCT for GAD has not been specifically evaluated in adolescents, which we therefore aimed to do in the current study.

**Methods:**

Ten adolescents with primary GAD were treated in a Norwegian Specialist Mental Health Care Setting by trained MCT therapists. Diagnoses were evaluated at pre- and post-treatment. Adolescent and parental self-report measures were used at pre- and post-treatment, and at 3- and 6-month follow-up. In addition, distress, metacognitive strategies and beliefs were reported on a sessional basis.

**Results:**

At post-treatment, nine out of 10 patients no longer met the diagnostic criteria for GAD, and several patients also recovered from comorbid disorders. The effect sizes from pre- to post-treatment on symptom measures and dysfunctional metacognitive beliefs were large and maintained at follow-up. Session-to-session descriptive data indicated a linear decrease in distress and metacognitive strategies and beliefs.

**Conclusion:**

These results provide preliminary evidence that MCT is associated with positive effects for adolescents with GAD and supports larger scale evaluations.

## Introduction

Generalized Anxiety disorder (GAD) is characterized by excessive worry about a variety of topics, accompanied by substantial emotional distress and symptoms such as sleep difficulties, concentration problems, exhaustion, irritability, and somatic symptoms (1). Disorder onset is typically in youth and the estimated prevalence in 14-year-olds is found to be as high as 4.5% (2). However, despite being prevalent and associated with high levels of functional impairment, adolescent GAD is substantially under-researched (3). As of now, the most common treatment option for adolescent GAD is cognitive behavioral therapy (CBT), where remission rates are only around 50% (4). A recent systematic review examining the efficacy of psychological therapies for GAD in children and adolescents highlights the need for further research into mechanisms of change and disorder-specific interventions for adolescent GAD (3).

In adults, Metacognitive therapy (MCT; 5) has shown to be a highly effective treatment for GAD, showing better outcomes when compared to other treatments in randomized controlled trials (6–8). MCT is founded on the Self-Regulatory Executive Function (S-REF) model (9), which emphasizes poor mental regulation caused by biases in metacognition as the key factor in persistent emotional distress and psychological dysfunction more generally.

In the metacognitive model of GAD (5, 10), two distinct types of worry contribute to distress and disorder. Type 1 worry concerns worry about non-cognitive events, such as disease/death, relationships or various negative experiences, while type 2 worry (i.e., *meta-worry*) refers to negative appraisals of worrying, for example thinking “what if my worrying never stops”. Both types of worry are linked to underlying dysfunctional metacognitive beliefs about worry. Positive metacognitive beliefs (e.g., “worrying helps me to solve problems”) are implicated in selecting type 1 worry as a coping strategy. Negative metacognitive beliefs about the uncontrollability and dangers of worry (e.g., “when I start worrying, I cannot stop”) prohibit disengagement from type 1 worry but also serve as the foundation for meta-worry/type 2 worry (5, 10). Hence, negative metacognitive beliefs are hypothesized as the most important mechanism for distress and contribute to maladaptive coping strategies such as avoidance or reassurance seeking in attempts to regulate worry. These strategies tend to backfire by increasing emotional distress and/or maintaining dysfunctional metacognitive beliefs. Consequently, MCT for GAD aims to modify biases in metacognition with an aim to remove meta-worry and improve mental regulation (i.e., reduce type 1 worry and maladaptive coping strategies), enabling adaptive top-down control and regulation of cognition (5).

To the best of the authors’ knowledge, no study has evaluated the effects associated with MCT in adolescents with GAD specifically or evaluated MCT when delivered individually to youths with GAD. However, one previous study of MCT adapted for children found the treatment to be associated with strong positive effects when delivered in a group format (11). The authors reported that 86% of the children (aged 7–13 years old) were free from their GAD diagnosis following treatment. Additionally, a recent trial of group MCT for children and adolescents with anxiety and depression (*N* = 97), where 53.6% of the participants were diagnosed with GAD at pre-treatment, found large reductions in total symptoms following treatment. Here, changes in metacognitive beliefs, dysfunctional strategies and perceived attention control were also identified as potential mechanisms of the treatment outcomes (12).

As these previous studies indicate that MCT might be a relevant treatment option for adolescent GAD, the current study set out to evaluate the effects associated with individual MCT for adolescent GAD (adhering to the treatment manual developed by 5) within a specialized mental health care setting, representing a naturalistic context. Independent assessors evaluated diagnoses at pre- and post-treatment, and self-report measures of emotional distress, metacognitive beliefs and well-being were also administered at 3- and 6 months follow-up. Additionally, session-to-session change patterns of distress and proposed metacognitive mechanisms were monitored during treatment.

We hypothesized that MCT for adolescent GAD would be associated with substantial improvement from pre-to post based on diagnostic assessment and self-report, and that these improvements should largely be maintained at follow-up. Additionally, we monitored session-to-session changes in proposed metacognitive mechanisms (e.g., worry and negative metacognitions) and expected improvements in them parallel to improvements in distress at the descriptive level.

## Methods

### Design and procedure

To evaluate MCT for adolescent GAD delivered in a naturalistic setting, we employed a pre-post design with 3- and 6-months follow-up. Ethical approval for this study was obtained by the Regional Committee for Medical and Health Research Ethics, Central Norway (Approval number: 533739). The research data was stored according to the general data protection rules and regulations at the hospital.

Adolescents diagnosed with primary GAD were recruited to the study from Child and Adolescent Psychiatry Outpatient Clinics in Middle-Norway. These services conduct diagnostic assessments based on several sources of information, including an anamnestic interview and a semi-structured diagnostic interview. Patients who met the diagnostic criteria for GAD were informed about the study and offered to be referred for assessment of eligibility but could also choose treatment as usual. For those referred to the study, the GAD diagnosis, potential secondary diagnoses, and an assessment of clinical severity of them, were re-evaluated by an independent assessor, using the criteria from the semi-structured interview: Anxiety Disorders Interview Schedule for DSM-IV: Child Version (ADIS-C; 13) followed by a diagnostic assessment in line with the current DSM-5 criteria (1). When a primary GAD diagnosis was confirmed, and the patient was deemed eligible for the study based on inclusion and exclusion criteria as described below, patients were asked if they were willing to participate and confirmed the terms by signing a form of consent (parents consented on their behalf when under the age of 16).

Patients over the age of 16 were asked whether their parents should or could be involved in the study and treatment, whilst parents were involved for patients under 16 years of age, in line with Norwegian law. The parents who participated actively in the treatment attended the pre- and post-assessment sessions together with their child, as well as completing parental self-reports. Additionally, they participated in an individual parental session during treatment, where they were informed about the treatment, given advice on relevant support for their adolescents (e.g., reassurance seeking interferes with your child discovering that worrying is not uncontrollable) and were able to ask questions.

Self-report measures were distributed before the first treatment session. With an aim to monitor progress in distress and the central metacognitive mechanisms over the course of therapy, a session-to-session assessment was administered at the beginning of every session.

Eligible patients were treated by three certified and trained MCT therapists who participated in weekly supervision to ensure adherence to the method.

Approximately one week after the last session, participants were re-assessed diagnostically by an independent assessor (typically the same person who conducted the assessment pre-treatment), and self-report was again administered. A measure of client satisfaction was administered to patients and participating parents at post-treatment.

Follow-up consisting of self-report was done at 3- and 6-months posttreatment. No MCT was administered between post-treatment and follow-up intervals. With an aim to achieve as complete data as possible, participants that completed the self-report battery at all time-points were offered a gift card (with a value of 500 NOK).

### Inclusion and exclusion criteria

The following inclusion/exclusion criteria were applied:

***Inclusion criteria:*** 1) Signed written informed consent obtained prior to entry in the study (only from parents when patients were under the age of 16); 2) Diagnosed with GAD (DSM-5, 1); 3) GAD considered the main cause of impairment and distress; 4) Between 13–17 years old at entry.

***Exclusion criteria*:** 1) Known somatic disease; 2) Psychosis; 3) Current suicide intent or ongoing and severe self-harming behavior; 4) Neurodevelopmental disorder; 5) Substance dependence; 6) Patients expected to initiate, discontinue or adjust psychotropic medication during treatment; 7) Severe conduct disorder; 8) Eating disorder.

### Participants

Initially, 19 participants were referred to the study (1 male and 18 females) of which 15 were deemed eligible based on the inclusion and exclusion criteria. Three out of the four that were not eligible had comorbid disorders that were part of our exclusion criteria, and the last was no longer interested in getting treatment for GAD. Out of the 15 that were originally included, five did not complete the treatment. One patient withdrew from the study after inclusion (following session 4) for unknown reasons, and one patient suddenly relocated due to private reasons (after session 2). Three patients were discontinued from the study by the research group due to emerging new information provided by the patients when a therapist got to know them better. This information indicated that GAD was not the primary presenting problem, and that they had psychiatric disorders and psychosocial needs with a higher priority (e.g., post-traumatic stress and eating disorder). Unfortunately, this information had not been identified in the assessment phase prior to referral to the study.

Thus, participants who completed treatment were 10 adolescent females aged 13 to 17 years (*M* = 15.20, *SD* = 1.48) with primary GAD. The distribution of participants` ages was as follows: 13 years old (*n* = 2), 14 years old (*n* = 1), 15 years old (*n* = 2), 16 years old (*n* = 3), 17 years old (*n* = 2). None of the participants were receiving psychotropic medications at the time of referral or during treatment. At pre-treatment, half of them were diagnosed with a comorbid anxiety disorder (see **Table 1**). All participants were of predominant Norwegian nationality and ethnicity. All the patients who completed treatment participated in follow-up at 3-months as well as at 6-months (*N* = 10).

**Table 1:** Overview of diagnoses based on the ADIS-C at pre-and post-treatment (*N* = 10).

Diagnoses	Pretreatment	Posttreatment
Generalized Anxiety Disorder	10	1
Panic Disorder	2	0
Agoraphobia	1	1
Obsessive-Compulsive Disorder	1	1
Social Anxiety Disorder	1	0

A total of seven participants (70%) included their parents in the treatment. Additionally, one set of parents provided self-report assessments even though they were not part of the treatment, bringing the total parent reports up to 80%.

### Measures

#### Diagnostic assessment and primary outcomes

The primary outcome was clinician-based assessment of the GAD diagnosis based on the DSM-5. Assessors made use of the ADIS-C in assessing the overall severity of symptoms and functional impairment, where a score of 4 or higher is considered indicative of clinically significant disorder. The ADIS-C is a widely used instrument for assessing treatment outcomes in clinical trials targeting childhood anxiety disorders (14, 15) with evidence of good reliability and validity for this purpose (16, 17).

#### Secondary outcomes

The battery of measures administered at pre-treatment, post-treatment and at follow-up were considered secondary outcomes and consisted of the following self-report measures (of which parents reported on the SCARED, SMFQ, and CSQ-8).

The Screen for Child Anxiety related Emotional Disorders (SCARED; 18, 19) is a widely used and validated measure for anxiety symptoms in both clinical and community samples (e.g. 20 & 21) and is available in both a child/adolescent and parent version. In this study we applied the version that consists of 41 items (e.g., “when I get frightened, I feel like passing out”, “I get stomachaches at school” and “I worry about other people liking me”). The total score ranging from 0 to 82 for both versions, where a higher score indicates more severe anxiety symptoms (18, 19).

The Short Mood and Feelings Questionnaire (SMFQ; 22) was used to assess depressive symptoms. The SMFQ is highly correlated with the original 33-item version, The Mood and Feelings Questionnaire (MFQ; 22), that is recommended for screening depression in children and adolescents by the National Institute for Health and Clinical Excellence 23). The SMFQ is available in both a child/adolescent and a parent version. It consists of 13 statements about the past two weeks (e.g., “I did everything wrong” or “I felt miserable or unhappy”) and the total score ranges from 0 to 26 for both versions, where a higher score indicates more depressive symptoms.

The Generalized Anxiety Disorder 7-item Scale (GAD-7; 24) was applied to self-rate severity and symptoms of generalized anxiety in this sample. The GAD-7 was originally developed for adults but has also been validated in adolescents (25). The GAD-7 enquires how often you have been bothered by problems such as “feeling nervous, anxious or on edge” or “worrying too much about different things” the past two weeks. These items are rated on a 4-point scale, ranging from no problem at all to problematic nearly every day. The total score ranges from 0 to 21, where higher scores indicate higher severity.

Penn State Worry Questionnaire- brief version (PSWQ-brief version) (26) is a self-report measure adapted from the original version of 16-items (27) measuring the tendency to worry. It is widely used and has been validated for use in adolescents (26) and consists of 5 items (e.g. “when I am under pressure, I worry a lot” or “I worry all the time”). We applied the brief version based on previous validation and to reduce the overall assessment burden for the adolescents in the study. The total score ranges from 5 to 25, where higher scores indicate a higher degree of worry proneness.

The Metacognitions Questionnaire-30 (MCQ-30; 28) assesses five different dimensions of metacognitive beliefs such as negative beliefs about uncontrollability and danger and positive beliefs about worry. Each item is rated on a 4-point Likert scale, from “Do not agree” to “Agree very much”. The measure has been validated in adolescents from 11 years old (29) as well as in older adolescents in Norway (30). The subscales range from 6 to 24, and the total score range from 30 to 120, where higher scores indicate a higher degree of dysfunctional metacognitive beliefs.

The Warwick-Edinburgh Mental Well-being Scale (WEMWBS; 31) measures mental wellbeing and covers both feeling and functional aspects of mental wellbeing. It is a widely applied measure found to have good psychometric properties in adolescents (32). The WEMWBS has 14 items (e.g. “I`ve been feeling optimistic about the future”, or “I`ve been feeling good about myself”) and is rated on a 5-point Likert scale from none of the time to all the time. The total score ranges from 14 to 70, where higher scores indicate greater mental wellbeing (31).

The Client Satisfaction Questionnaire (CSQ-8; 33) is an 8-item measure of client satisfaction of services with both a parent and a child version. Participants rated items such as overall satisfaction, perceived quality of services, whether their needs were met and if they would recommend the service to others on a 4 point-scale from “very satisfied” to “very dissatisfied”. The total score ranges from 8 to 32 for both versions, where higher scores indicate higher satisfaction with treatment.

Session-to-Session Assessment was conducted with visual analogue scales (VAS) based on the Generalized Anxiety Disorder – Self-Rating (GADS-R; 5) to monitor distress/anxiety, levels of worry during the last week, and endorsement of negative- and positive metacognitions. The primary intention of the measure was to inform clinicians about changes on a weekly basis throughout therapy, which can be helpful in order to identify therapeutic targets. Level of distress and the use of worry was rated from 0 (“not problematic”) to 8 (“very problematic”). Furthermore, the negative metacognitive belief that worrying is uncontrollable and the positive metacognitive belief that worrying is advantageous were rated from 0% (“not true”) to 100% (“completely true”).

### Treatment protocol

The full treatment protocol is outlined in Wells (5), originally developed for the treatment of adult GAD. The treatment consists of creating an idiosyncratic case-formulation which is used to socialize the patient to the metacognitive perspective. Introducing and practicing *detached mindfulness*, as an alternative mental state in response to trigger thoughts, is a key component aiming to reduce maladaptive metacognitive strategies (e.g., worry, reassurance seeking, avoidance) and to modify dysfunctional metacognitive beliefs. Metacognitive beliefs are evaluated and tested through verbal restructuring and behavioral experiments. Towards the end of therapy, an old-new plan is made to consolidate what is learned in treatment.

In the current study, we aimed to follow the original treatment protocol (5) and allowed for up to 12 sessions, similar to a randomized controlled trial conducted with adults (6). The formulation used, treatment sequence, core techniques etc. were exactly like adult treatment. Relevant adjustments related to working with adolescents were for example simplification of language when needed, using personally relevant and age-adjusted metaphors when introducing detached mindfulness (e.g., “have you ever ignored your mother calling for you, and continued what you were doing? Is it possible to respond to trigger thoughts the same way?”), more repetition of central concepts/techniques, and writing down homework assignments. Time was devoted to parental involvement when appropriate, typically as part of the initial socialization phase (e.g., help parents understand that reassurance seeking, reasoning with thoughts, positive thinking, etc., can block metacognitive belief change), and in aiding with experiments (e.g., test danger beliefs and worry modulation experiments).

### Statistical analyses

Analyses were performed in STATA IC version 19.5. Differences in symptoms over time were assessed using planned contrast within multilevel modelling, which allows for estimation of changes in repeated measures over time despite missing data.

Analyses were conducted using linear mixed-effects models estimated via restricted maximum likelihood (REML). This approach allows inclusion of participants with partially missing repeated measures under the assumption that data are missing at random (MAR). We assessed the assumption of missing completely at random (MCAR) using Little’s test *χ^2^*(6) = 5.95, *p* = .43. The test indicated that missingness was not systematically related to observed data, supporting the use of likelihood-based mixed models under the assumption of missing at random (MAR). Thus, all available observations contributed to parameter estimation.

For model comparison, maximum likelihood (ML) estimation was used. In the random coefficients model for secondary outcomes, we investigated the extent to which, after having received treatment, the gains were sustained or improved at 3- and 6-months follow-up. We computed a time effect, representing the difference in the change over time for the group. The linear combination of coefficients function was used to create model-predicted means and all planned contrasts. Parameter estimates and confidence intervals are reported based on restricted maximum likelihood (REML), whereas model comparisons were conducted using maximum likelihood (ML) estimation to ensure valid likelihood-based comparisons across models with different fixed effects.

The observed means and SD values as well as the model predicted means and SE values are presented for continuous variables at all time-points from pre-treatment to follow-up. We also calculated within-group effect sizes using Hedges´ *g* based on completers.

As independent clinical assessment was restricted to pre- and post-treatment, we used the GAD-7 to assess clinical recovery rates and categories of remission, no change or worsened. Clinical reliable change was estimated according to Jacobsen and Truax (34). The GAD-7 is often used to indicate clinical cut-off scores, where a score of ≥ 10 can refer a clinical level of symptoms (24, 35, 36). Reliable change in the GAD-7 was defined as a change of 4 points or more (e.g. 37).

## Results

### Treatment outcomes

**Table 1** presents the diagnoses pre- and post-treatment as evaluated by the independent assessors using the ADIS-C (13). One out of ten patients met the diagnostic criteria for GAD following treatment. In the whole sample, a total of 15 diagnoses were identified at pre-treatment, where five individuals met the criteria for one additional diagnosis. At post-treatment, a total of three diagnoses remained in the sample.

For the secondary outcomes, using the planned contrast in the multilevel model analysis showed that all included measures show a significant improvement from pre- to post-treatment (**Table 2**). The effect sizes (*g*) for patient self-reports were large, indicating a substantial change from pre- to post-treatment. Parents also reported substantial improvements from pre- to post-treatment, indicated by a large effect size for anxiety symptoms and a moderate effect size for depression symptoms. Note that eight patients had parents involved in reporting symptoms which constitute the foundation for the analyses.

**Table 2:** Observed and model predicted means showing difference in change over time for primary outcome from pre- to post-treatment in the total sample *(N* = 10).

Observed means		Model predicted means		Change		Hedges` *g*
Pre-treatment (*sd*)	Post-treatment (*sd*)	Pre-treatment (se)	Post-treatment (se)	Difference in change over time Z-value (p-value)	Time effect 95% CI	
Scared_c						
52.60 (7.86)	25.00 (12.86)	52.60 (2.49)	25.00 (4.07)	-7.01 (.00)	[-35.31, -19.89]	2.03
Scared_p						
42.25 (14.75)	25.63 (10.18)	42.25 (4.48)	25.63 (4.48)	-2.62 (.01)	[-29.05, -4.20]	.80
SMFQ_c						
14.00 (4.03)	5.50 (3.92)	14.00 (1.26)	5.50 (1.26)	-9.74 (.00)	[-10.21, -6.79]	2.81
SMFQ_p						
10.38 (6.95)	4.38 (3.89)	10.38 (1.99)	4.38 (1.99)	-2.13 (.05)	[-11.52, -.48]	.61
PSWQ						
22.90 (2.23)	14.80 (4.10)	22.90 (.71)	14.80 (1.27)	-7.65 (.00)	[-10.17, -6.02]	2.21
MCQ-30						
73.80 (8.83)	46.60 (11.72)	73.80 (2.79)	46.60 (3.59)	-12.04 (.00)	[-31.63, -22.77]	3.48
MCQ neg						
18.40 (3.37)	10.70 (2.50)	18.40 (.94)	10.70 (.94)	-6.96 (.00)	[-9.76, -5.64]	3.83
MCQ pos						
11.50 (3.54)	7.20 (3.12)	11.50 (1.05)	7.20 (1.05)	-5.26 (.00)	[-5.90, -2.70]	2.83
GAD7						
16.20 (2.74)	7.20 (5.59)	16.20 (.87)	7.20 (1.60)	-6.67 (.00)	[-11.65, -6.35]	1.93
Wemwbs						
37.50 (4.90)	48.70 (8.84)	37.50 (1.55)	48.70 (2.80)	4.71 (.00)	[6.54, 15.86]	-2.18

SCARED, The Screen for Child Anxiety related Emotional Disorders (c = child, p = parent); SMFQ, Short version of the Mood and Feelings Questionnaire (c =child, p = parent); PSWQ, Penn State Worry Questionnaire-brief version; MCQ-30, Metacognitions questionnaire-30; MCQ neg, Metacognitions questionnaire negative beliefs; MCQ pos, Metacognitions questionnaire positive beliefs; GAD-7, Generalized Anxiety Disorder Scale; WEMWBS, The Warwich-Edinburgh Mental Wellbeing Scale.

The multilevel model analyses furthermore revealed non-significant changes over time from post-treatment, through 3-months and to 6-months follow-up, indicating longer term stability in the effects achieved from pre- to post-treatment (**Table 3**).

**Table 3:** Observed and model predicted means showing difference in change over time for secondary outcome from post-treatment to 3- and 6-months follow-up in the total sample (*N* = 10).

Observed means			Model predicted means bootstrapped			Change	
Post-treatment (*sd*)	3-months follow-up (*sd*)	6-months follow-up (sd)	Post-treatment (se)	3-months follow-up (se)	6-months follow-up (se)	Diff. in change over time, Z-value (p-value)	Time effect 95% CI
Scared_c							
25.00 (12.86)	29.20 (15.04)	27.00 (15.24)	25.00 (4.22)	29.20 (4.35)	27.00 (4.73)	.58 (.56)	[-2.37, 4.37]
Scared_p							
25.63 (10.18)	27.75 (12.17)	19.63 (19.23)	25.63 (3.79)	27.75 (3.83)	19.63 (3.94)	-1.39 (0.16)	[-7.21, 1.22]
SMFQ_c							
5.50 (3.92)	8.89 (7.16)	6.30 (3.86)	5.50 (1.65)	8.80 (1.65)	6.30 (1.65)	.46 (.65)	[-1.31, 2.11]
SMFQ_p							
4.38 (3.89)	5.75 (6.09)	5.13 (4.97)	4.38 (1.72)	5.75 (1.76)	5.13 (1.87)	.33 (.74)	[-1.82, 2.57]
PSWQ							
14.80 (4.10)	15.30 (5.25)	16.00 (4.71)	14.80 (1.49)	15.30 (1.49)	16.00 (1.49)	1.02 (.31)	[-.55, 1.75]
MCQ-30							
46.60 (11.72)	50.40 (13.30)	48.50 (13.52)	46.60 (4.07)	50.40 (4.07)	48.50 (4.07)	.70 (.48)	[-1.69, 3.59]
MCQ neg							
10.70 (2.50)	11.50 (5.00)	11.40 (4.27)	10.70 (1.11)	11.50 (1.17)	11.4 (1.35)	.60 (.55)	[-.79, 1.49]
MCQ pos							
7.20 (3.12)	8.30 (2.95)	7.30 (1.57)	7.20 (.83)	8.30 (.83)	7.3 (.83)	.14 (.89)	[-.66,.76]
GAD7							
7.20 (5.59)	8.89 (4.89)	7.70 (4.62)	7.20 (1.60)	8.80 (1.60)	7.70 (1.60)	.49 (.62)	[-.75, 1.25]
Wemwbs							
48.70 (8.84)	42.80 (8.84)	49.50 (6.13)	48.70 (2.54)	42.80 (2.54)	49.50 (2.54)	.25 (.81)	[-2.78, 3.58]

SCARED, The Screen for Child Anxiety related Emotional Disorders (c = child, p = parent); SMFQ, Short version of the Mood and Feelings Questionnaire (c =child, p = parent); PSWQ, Penn State Worry Questionnaire-brief version; MCQ-30, Metacognitions questionnaire-30; MCQ neg, Metacognitions questionnaire negative beliefs; MCQ pos, Metacognitions questionnaire positive beliefs; GAD-7, Generalized Anxiety Disorder Scale; WEMWBS, The Warwich-Edinburgh Mental Wellbeing Scale.

### Clinically significant change

As clinical assessment was restricted to pre- and post-treatment, we used the GAD-7 to classify participants as recovered, improved, no change, or worsened comparing their post-, 3-months, and 6-months follow-up scores to their pre-score. Based on this measure, the treatment was associated with a 70-90% recovery rate. None were classified as worsened at any time point. The specific classifications are presented in **Table 4**.

**Table 4:** Clinically significant change for completers on primary outcomes from pre- to post-treatment (*N* = 10).

Timepoint	Recovered	Improved	No change	Worsened
Post-treatment	8 (80%)	1 (10%)	1 (10%)	0 (0%)
3 months follow-up	7 (70%)	1 (10%)	2 (20%)	0 (0%)
6 months follow-up	9 (90%)	0 (0%)	1 (10%)	0 (0%)

### Session to session change

**Figure 1:**
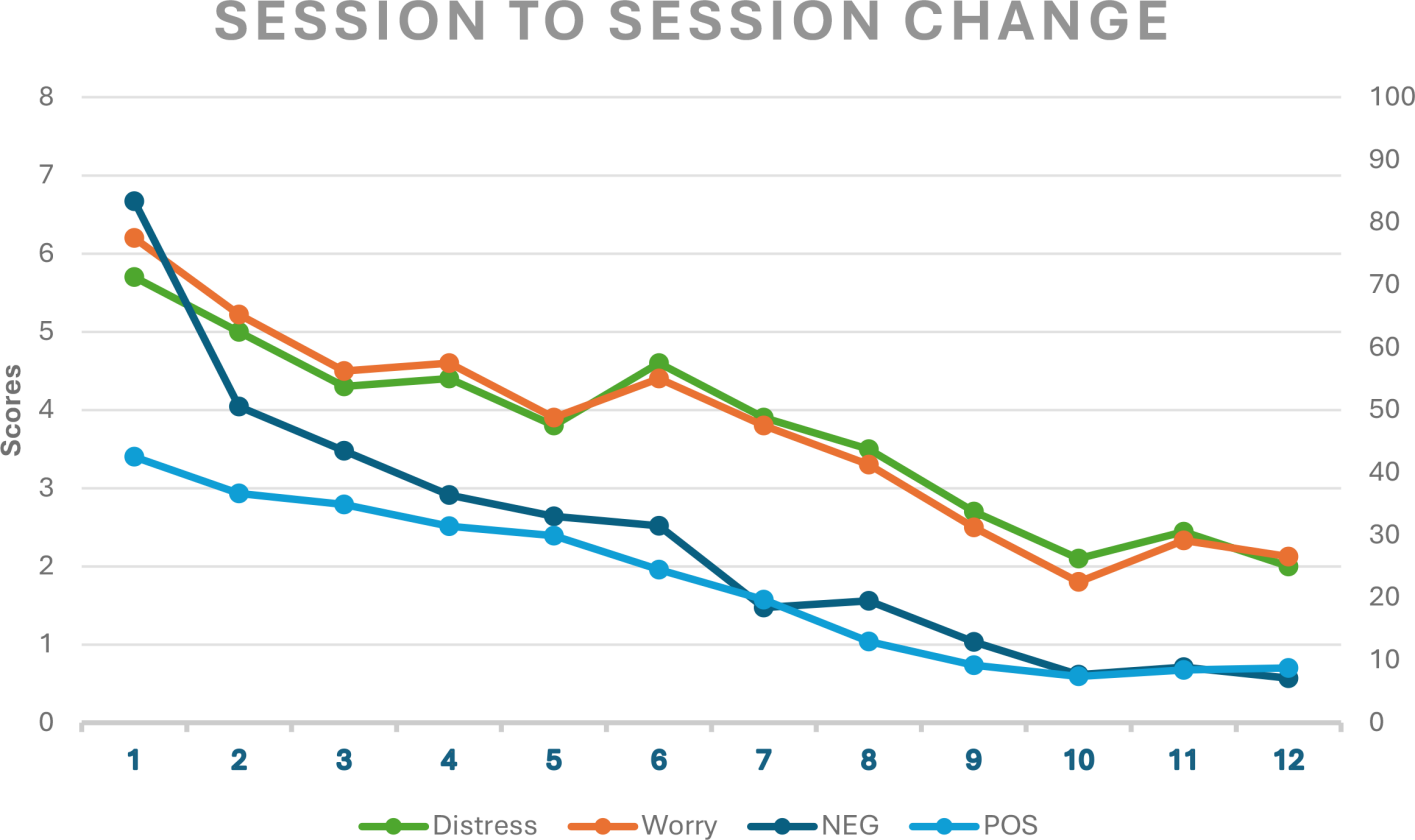
Session to session change mechanisms related to distress, worry, negative beliefs and positive beliefs measured from session 1 through 12 (*N* = 10). NEG, Negative metacognitive beliefs about the uncontrollability of worrying; POS, Positive metacognitive beliefs of the usefulness of worry. The vertical axis on the left is rated from 0–8 and captures distress (green) and worry (orange), the vertical axis on the right is measured from 0–100 and captures NEG (dark blue) and POS (light blue). The horizontal axis represents session 1-12.

At the beginning of every session, we monitored distress and metacognitive mechanisms based on VAS-items informed by the GADs-R (5). As displayed in **Figure 1**, distress, worrying, and positive- and negative metacognitive beliefs (uncontrollability) decreased over the course of treatment in the total sample.

### Client satisfaction

To assess satisfaction with treatment, we administered the Client Satisfaction Questionnaire (CSQ-8; 33) to patients (*N* = 10) and parents (*N* = 8) separately following treatment. The mean score for patients in our sample was 26.80 (*SD* = 2.39) and the mean score for parents was 28.50 (*SD* = 3.07), indicating that the overall level of treatment satisfaction was high for both patients and parents.

## Discussion

In the current study, we evaluated the effects associated with metacognitive therapy for adolescent GAD, delivered individually and in a specialist mental health care setting.

Nine (90%) out of the ten participants treated no longer met the diagnostic criteria for GAD at post-treatment assessment. In addition, among the five who presented with comorbid disorders at pre-treatment, three (60%) were free of comorbid disorders at post-treatment. Based on self-report, the treatment was associated with substantial improvements marked by large effect sizes from pre- to post-treatment on measures of anxiety symptoms, depression, worry proneness, mental well-being and dysfunctional metacognitions. Parent reports also reported substantial improvements consistent with large effect sizes on anxiety symptoms and a moderate effect size on depression symptoms. Both patients and parents reported high treatment satisfaction. Based on clinical reliable change assessments using the GAD-7, eight patients (80%) were classified as recovered, one as improved (10%), and one (10%) as unchanged at post-treatment.

Treatment gains were largely maintained at follow-up as the multilevel model indicated non-significant changes from post-treatment, through 3-months and to 6-months follow-up. This observation was also supported by clinically reliable change classification where seven (70%) classified as recovered, one (10%) as improved, and two (20%) as unchanged at 3-months, and nine (90%) classified as recovered, and one (10%) as unchanged at 6-months.

Monitoring sessional changes in distress, time spent worrying the last week, and positive- and negative metacognitions indicated a linear decrease throughout the treatment in the total sample. A steep decrease in negative metacognitive beliefs about the uncontrollability of worry was observed in early phase of the treatment.

It should be noted that five patients that were initially included in the study did not complete the treatment. One patient withdrew from the treatment for unknown reasons. Another patient suddenly relocated due to private reasons. Three patients showed emerging difficulties related to distress and more serious disorders that had not been revealed during the assessment phase (e.g., symptoms consistent with traumatic experiences and post-traumatic stress disorder that needed to be evaluated; eating disorder). Their primary disorder was thus not GAD. While pre-data from the one patient that relocated could have been included in the analysis, we choose to focus on those that participated in the treatment considering the small sample and the open design.

Overall, the results are in line with our hypothesis and provide preliminary evidence for positive effects associated with applying MCT to adolescents with GAD. Consistent with the metacognitive model (5, 38) MCT was also associated with transdiagnostic effects at the disorder and symptoms level. This result is also consistent with a previous study that has evaluated the effects of group MCT for children (11), and a study evaluating the effects associated with group MCT in youths with common mental disorders including a large proportion of patients with GAD (12).

In line with the proposed metacognitive mechanisms of GAD (10) and key therapeutic targets in MCT for GAD (5), our patients reported a substantial reduction in their endorsement of dysfunctional metacognitive beliefs associated with the treatment. This observation supports metacognitive change as potentially important for symptom improvement among adolescents and is in line with empirical research on the mechanistic role for metacognitive change to symptom improvements in both adults (see 39 for a review) and youths (12). More studies in adolescents testing metacognitive mechanisms more rigorously in larger trials and with better designs should be conducted to draw firm conclusions.

The present study is associated with several limitations that must be acknowledged. The open-trial design without a control group impedes inferences regarding the associations observed and their link to the treatment. The authors of the current manuscript served as the therapists in the trial, which is associated with researcher bias. The sample size was limited, included only female patients, and relatively homogenous in terms of for example ethnicity, which limits generalizability. Session to session changes were descriptively reported due to the low sample size and can only suggest co-occurring reductions in mechanisms and distress. Strengths of the study include using independent assessors of diagnoses pre- and post-treatment, trained MCT therapists, and the natural treatment setting. As pointed out by Shipp et al. (3), existing treatment research on adolescent GAD is very limited, so in that regard our study is important and suggests that MCT and its mechanisms should be investigated further and with more stringent research designs.

In conclusion, these results indicate that MCT for GAD can be applied to adolescents with adjustments mainly related to the delivery of treatment. In our study, formulation, sequence and core techniques from the adult treatment were maintained while adjustments comprised for example modification of language, choice of examples/metaphors, and parental involvement. The treatment was associated with positive effects at the diagnosis and symptom level, including improvements in comorbid disorders and secondary outcomes. These associations were largely maintained at 3- and 6-months follow-up. The improvements were observed parallel to metacognitive change, suggesting that these mechanisms are interesting to pursue in future research. In sum, our preliminary results support the metacognitive approach as a promising treatment option for adolescents with GAD.

## Data Availability

The datasets presented in this article are not readily available because Data is confidential. Requests to access the datasets should be directed to hanne.undheim@ntnu.no.

## References

[1] American Psychiatric Association . Diagnostic and statistical manual of mental disorders (5th ed). American Psychiatric Publishing. (2013). doi: 10.1176/appi.books.9780890425596.

[2] Steinsbekk S Ranum B Wichstrøm L . Prevalence and course of anxiety disorders and symptoms from preschool to adolescence: A 6-wave community study. J Child Psychol Psychiatry. (2022) 63:527–34. doi: 10.1111/jcpp.13487. PMID: 34318492

[3] Shipp L Leigh E Rajesh S Waite P . Evaluating the efficacy of psychological therapies for generalised anxiety disorder in children and adolescents: A systematic review and narrative synthesis. JCPP Adv. (2025) 5:e70056. doi: 10.1002/jcv2.70056. PMID: 41815761 PMC12973129

[4] James AC Reardon T Soler A James G Creswell C . Cognitive behavioural therapy for anxiety disorders in children and adolescents. Cochrane Database Systematic Rev. (2020) 11:CD013162. doi: 10.1002/14651858.CD013162.pub2. PMID: 33196111 PMC8092480

[5] Wells A . Metacognitive Therapy for Anxiety and Depression. New York: Guilford press (2009).

[6] Nordahl HM Borkovec TD Hagen R Kennair LEO Hjemdal O Solem S . Metacognitive therapy versus cognitive–behavioural therapy in adults with generalised anxiety disorder. Br J Psychiatry Open. (2018) 4:393–400. doi: 10.1192/bjo.2018.54. PMID: 30294448 PMC6171331

[7] van der Heiden C Muris P van der Molen HT . Randomized controlled trial on the effectiveness of metacognitive therapy and intolerance-of-uncertainty therapy for generalized anxiety disorder. Behav Res Ther. (2012) 50:100–9. doi: 10.1016/j.brat.2011.12.005. PMID: 22222208

[8] Wells A Welford M King P Papageorgiou C Wisely J Mendel E . A pilot randomized trial of metacognitive therapy vs applied relaxation in the treatment of adults with generalized anxiety disorder. Behav Res Ther. (2010) 48:429–34. doi: 10.1016/j.brat.2009.11.013. PMID: 20060517

[9] Wells A Matthews G . Attention and emotion: A clinical perspective. Lawrence Erlbaum Associates, Inc (1994).

[10] Wells A . Meta-cognition and worry: A cognitive model of generalized anxiety disorder. Behav Cogn Psychother. (1995) 23:301–20. doi: 10.1017/S1352465800015897. PMID: 41292463

[11] Esbjørn BH Normann N Christiansen BM Reinholdt-Dunne ML . The efficacy of group metacognitive therapy for children (MCT-c) with generalized anxiety disorder: An open trial. J Anxiety Disord. (2018) 53:16–21. doi: 10.1016/j.janxdis.2017.11.002. PMID: 29145078

[12] Thingbak A Wells A O’Toole MS . Group metacognitive therapy for children and adolescents with anxiety and depression: A preliminary trial and test of proposed mechanisms. J Anxiety Disord. (2024) 107:102926. doi: 10.1016/j.janxdis.2024.102926. PMID: 39321673

[13] Silverman WK Albano AM . Anxiety Disorders Interview Schedule for DSM-IV: Child Version. New York: Graywind Publications (1996).

[14] Ginsburg GS Kendall PC Sakolsky D Compton SN Piacentini J Albano AM . Remission after acute treatment in children and adolescents with anxiety disorders: Findings from the CAMS. J Consulting Clin Psychol. (2011) 79:806–13. doi: 10.1037/a0025933 PMC3371083 22122292

[15] Hudson JL Newall C Rapee RM Lyneham HJ Schniering CC Wuthrich VM . The impact of brief parental anxiety management on child anxiety treatment outcomes: A controlled trial. J Clin Child Adolesc Psychol. (2014) 43:370–80. doi: 10.1080/15374416.2013.807734. PMID: 23845064 PMC4037847

[16] Silverman WK Saavedra LM Pina AA . Test-retest reliability of anxiety symptoms and diagnoses with the Anxiety Disorders Interview Schedule for DSM-IV: Child and parent versions. J Am Acad Child Adolesc Psychiatry. (2001) 40:937–44. doi: 10.1097/00004583-200108000-00016. PMID: 11501694

[17] Wood JJ Piacentini JC Bergman RL McCracken J Barrios V . Concurrent validity of the anxiety disorders section of the Anxiety Disorders Interview Schedule for DSM-IV: Child and parent versions. J Clin Child Adolesc Psychol. (2002) 31:335–42. doi: 10.1207/S15374424JCCP3103_05. PMID: 12149971

[18] Birmaher B Khetarpal S Brent D Cully M Balach L Kaufman J . The Screen for Child Anxiety Related Emotional Disorders (SCARED): Scale construction and psychometric characteristics. J Am Acad Child Adolesc Psychiatry. (1997) 36:545–53. doi: 10.1097/00004583-199704000-00018. PMID: 9100430

[19] Birmaher B Brent DA Chiappetta L Bridge J Monga S Baugher M . Psychometric properties of the Screen for Child Anxiety Related Emotional Disorders (SCARED): A replication study. J Am Acad Child Adolesc Psychiatry. (1999) 38:1230–6. doi: 10.1097/00004583-199910000-00011. PMID: 10517055

[20] Leikanger E Ingul JM Larsson B . Sex and age-related anxiety in a community sample of Norwegian adolescents. Scandinavian J Psychol. (2012) 53:150–7. doi: 10.1111/j.1467-9450.2011.00915.x. PMID: 22022875

[21] Weitkamp K Romer G Rosenthal S Wiegand-Grefe S Daniels J . German Screen for Child Anxiety Related Emotional Disorders (SCARED): Reliability, validity, and cross-informant agreement in a clinical sample. Child Adolesc Psychiatry Ment Health. (2010) 4:19. doi: 10.1186/1753-2000-4-19. PMID: 20591137 PMC2912250

[22] Angold A Costello EJ Messer SC Pickles A Winder F Silver D . The development of a short questionnaire for use in epidemiological studies of depression in children and adolescents. Int J Methods Psychiatr Res. (1995) 5:237–49. doi: 10.1002/mpr.47. PMID: 41531421

[23] National Institute for Health and Care Excellence . Depression in children and young people: identification and management (2019). Available online at: https://www.nice.org.uk/guidance/ng134 (Accessed November 10, 2025). 31577402

[24] Spitzer RL Kroenke K Williams JBW Löwe B . A brief measure for assessing generalized anxiety disorder: The GAD-7. Arch Internal Med. (2006) 166:1092–7. doi: 10.1001/archinte.166.10.1092. PMID: 16717171

[25] Mossman SA Luft MJ Schroeder HK Varney ST Fleck DE Barzman DH . The Generalized Anxiety Disorder 7-item scale in adolescents with generalized anxiety disorder: Signal detection and validation. Ann Clin Psychiatry. (2017) 29:227–234A. doi: 10.12788/acp.0027. PMID: 29069107 PMC5765270

[26] Topper M Emmelkamp PM Watkins E Ehring T . Development and assessment of brief versions of the Penn State Worry Questionnaire and the Ruminative Response Scale. Br J Clin Psychol. (2014) 53:402–21. doi: 10.1111/bjc.12052. PMID: 24799256

[27] Meyer TJ Miller ML Metzger RL Borkovec TD . Development and validation of the penn state worry questionnaire. Behav Res Ther. (1990) 28:487–95. doi: 10.1016/0005-7967(90)90135-6. PMID: 2076086

[28] Wells A Cartwright-Hatton S . A short form of the metacognitions questionnaire: Properties of the MCQ-30. Behav Res Ther. (2004) 42:385–96. doi: 10.1016/s0005-7967(03)00147-5. PMID: 14998733

[29] Li F Yuan D Gao C Xiong K Geng F Zhang L . Validity and reliability of the Metacognitions Questionnaire-30 (MCQ-30) among Chinese adolescents. Child Psychiatry Hum Dev. (2023) 54:1526–38. doi: 10.1007/s10578-023-01625-7. PMID: 37917240

[30] HoffHU HjemdalO SteinsbekkS NordahlH . Psychometric properties of the Metacognitions Questionnaire-30 (MCQ-30) in older Norwegian adolescents. Child Psychiatry Hum Dev. (2025). doi: 10.1007/s10578-025-01843-1. PMID: 40266510

[31] Tennant R Hiller L Fishwick R Platt S Joseph S Weich S . The warwick–edinburgh mental well-being scale (WEMWBS): development and UK validation. Health Qual Life Outcomes. (2007) 5:63. doi: 10.1186/1477-7525-5-63. PMID: 18042300 PMC2222612

[32] McKay MT Andretta JR . Evidence for the psychometric validity, internal consistency and measurement invariance of Warwick-Edinburgh Mental Well-being Scale scores in Scottish and Irish adolescents. Psychiatry Res. (2017) 255:382–6. doi: 10.1016/j.psychres.2017.06.071. PMID: 28666244

[33] Attkisson CC Zwick R . The Client Satisfaction Questionnaire: Psychometric properties and correlations with service utilization and psychotherapy outcome. Eval Program Plann. (1982) 5:233–7. doi: 10.1016/0149-7189(82)90074-X 10259963

[34] Jacobson NS Truax P . Clinical significance: A statistical approach to defining meaningful change in psychotherapy research. J Consulting Clin Psychol. (1991) 59:12–9. doi: 10.1037/0022-006X.59.1.12. PMID: 2002127

[35] Kroenke K Spitzer RL Williams JBW Monahan PO Löwe B . Anxiety disorders in primary care: Prevalence, impairment, comorbidity, and detection. Ann Internal Med. (2007) 146:317–25. doi: 10.7326/0003-4819-146-5-200703060-00004. PMID: 17339617

[36] Tiirikainen K Haravuori H Ranta K Kaltiala-Heino R Marttunen M . Psychometric properties of the 7-item Generalized Anxiety Disorder Scale (GAD-7) in a large representative sample of Finnish adolescents. Psychiatry Res. (2019) 272:30–5. doi: 10.1016/j.psychres.2018.12.004. PMID: 30579178

[37] Gyani A Shafran R Layard R Clark DM . Enhancing recovery rates: Lessons from year one of IAPT. Behav Res Ther. (2013) 51:597–606. doi: 10.1016/j.brat.2013.06.004. PMID: 23872702 PMC3776229

[38] Wells A . Breaking the cybernetic code: Understanding and treating the human metacognitive control system to enhance mental health. Front Psychol. (2019) 10:2621. doi: 10.3389/fpsyg.2019.02621. PMID: 31920769 PMC6920120

[39] Pukstad E Halvorsen JØ Jensen MR Solhaug S Nordahl H . Do metacognitive beliefs satisfy criteria as mechanisms of change in treatment? A systematic review and evidence synthesis. Clin Psychol Rev. (2025) 122:102121. doi: 10.1016/j.cpr.2025.102654. PMID: 41056592

